# A Case of MEN 2A Syndrome With Bilateral Recurrent Pheochromocytoma: A Case Report and Literature Review

**DOI:** 10.7759/cureus.116229

**Published:** 2026-09-14

**Authors:** Shoag Albugami, Hussam A Alharbi, Khalid M Alhajri, Khaled Altoukhi, Layan Alrasheed

**Affiliations:** 1 General Surgery, Prince Sultan Military Medical City, Riyadh, SAU; 2 General Surgery - Breast and Endocrine Surgery Unit, Al Kharj Armed Forces Hospital, Riyadh, SAU; 3 General Surgery - Breast and Endocrine Surgery, Prince Sultan Military Medical City, Riyadh, SAU; 4 General Surgery, Specialized Medical Center, Riyadh, SAU; 5 Surgical Oncology, Upper GI, and General Surgery, Prince Sultan Military Medical City, Riyadh, SAU; 6 Endocrinology, Prince Sultan Military Medical City, Riyadh, SAU

**Keywords:** malignant pheochromocytoma, medullary thyroid carcinoma (mtc), men 2a, men syndrome, pheochromocytoma

## Abstract

Pheochromocytoma is a neuroendocrine tumor arising from chromaffin cells and producing catecholamines. Due to the excessive release of catecholamines, these tumors can present with paroxysmal episodes of high blood pressure, headache, diaphoresis, and palpitations. We present the case of a 23-year-old female patient who presented to the clinic with a history of headache, palpitations, and vomiting for two months. She was admitted for further investigations and management. Laboratory studies revealed elevated urinary and plasma catecholamine levels. Further work-up revealed bilateral adrenal masses diagnosed as pheochromocytoma and medullary thyroid cancer. The patient was diagnosed with multiple endocrine neoplasia (MEN) type 2 and underwent bilateral laparoscopic adrenalectomy and total thyroidectomy with central and lateral neck dissection, and during surveillance, she was found to have recurrent pheochromocytoma. We also discuss the diagnosis and management of recurrent pheochromocytoma in MEN syndrome.

## Introduction

Pheochromocytoma is a neuroendocrine tumor arising from chromaffin cells and producing catecholamines [1,2]. Due to the excess of released catecholamines, these tumors can present with paroxysmal episodes of high blood pressure, headache, diaphoresis, and palpitations [1,2]. Most pheochromocytomas originate from the adrenal gland’s medulla. Pheochromocytoma is known as the “10% tumor,” with 10% being extra-adrenal, 10% familial, 10% bilateral, 10% pediatric, and 10% malignant [1,3]. The diagnosis of pheochromocytoma is made by biochemical and radiological investigations. Urine or serum catecholamines (normetanephrine and metanephrine) and imaging studies including MRI, CT imaging, and ^131^I metaiodobenzylguanidine (MIBG) scanning, or FDG PET scanning help confirm the diagnosis [3,4]. Familial pheochromocytoma usually occurs with autosomal dominant inheritance, such as von Hippel-Lindau (VHL) syndrome and multiple endocrine neoplasia type 2 (MEN 2), and is the first manifestation in 50% of patients with VHL disease and in 27% of patients with MEN 2A syndrome [5].

Here we present a case of a 23-year-old female patient who presented to the hospital with the classic presentation of pheochromocytoma and was diagnosed later with MEN 2A syndrome.

This article was previously presented as an oral presentation at the 2024 Saudi Society of Breast and Endocrine Conference, Riyadh, Saudi Arabia.

## Case presentation

We present a case of a 23-year-old female patient, not known to have any medical illness, who was referred to our hospital with a two-month history of throbbing headache, moderate in severity, associated with palpitations, sweating, nausea, and vomiting. Past medical and surgical history was unremarkable. Upon presentation, she was sitting comfortably on the bed, not in pain or respiratory distress, and was conscious and alert. Her vital signs were as follows: temperature of 37.3°C, heart rate of 88 beats per minute, respiratory rate of 21 breaths per minute, and blood pressure of 178/97 mmHg. The physical examination was unremarkable, except for acanthosis nigricans. The laboratory investigations are given in Table 1.

**Table 1:** The laboratory investigations

Lab test	Result	Reference
WBC	11	4-11x10^9^/L (HI)
Hemoglobin	145	115-165 g/L
platelets	583	150-450x10^9^/L
Creatinine	76	45-84 mcmol/L
Potassium	4.4	3.5-5.2 mmol/L
Sodium	135 mmol/L	135-145 mmol/L
Plasma metanephrine	+1,670 ng/L	<70 ng/L
Plasma normetanephrine	+682 ng/L	<120 ng/L
Urine 24-hour collection of metanephrine	68.95	<2.04 umol/24hrs
Urine 24-hour collection of normetanephrine	19.50	<4.04 umol/24hrs
Metanephrine/creatinine ratio	+8539	14-31 ug/g
Noradrenaline/creatinine ratio	+2239	47-310 ug/g

She was admitted under the care of the Breast and Endocrine Surgery Department in Prince Sultan Military Medical City, Riyadh, Saudi Arabia, for further investigations and management. CT of the abdomen with IV contrast was performed, which showed right adrenal mass measuring 4.2 x 3.6 cm having absolute washout of 50% (unenhanced: 38 HU, portovenous: 92 HU, delayed: 65 HU). It abutted but did not invade the IVC. The left mass measured 5 x 3.8 cm with absolute washout of 58% (unenhanced: 36 HU, portovenous: 48 HU, delayed: 41 HU), with necrosis posterolaterally (Figures 1, 2).

**Figure 1:**
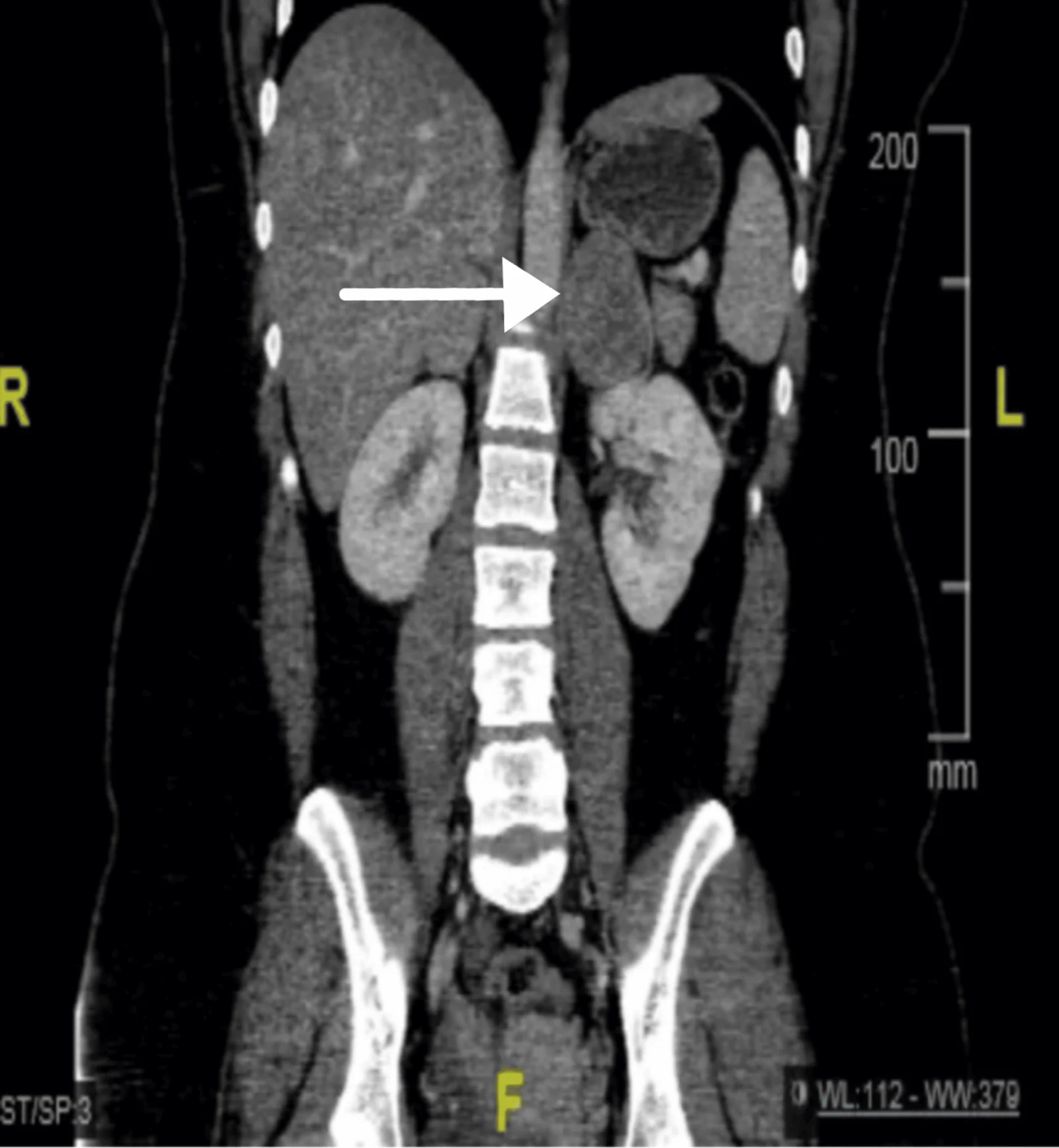
Coronal CT view of the abdomen with IV contrast (the arrow points to the mass)

**Figure 2:**
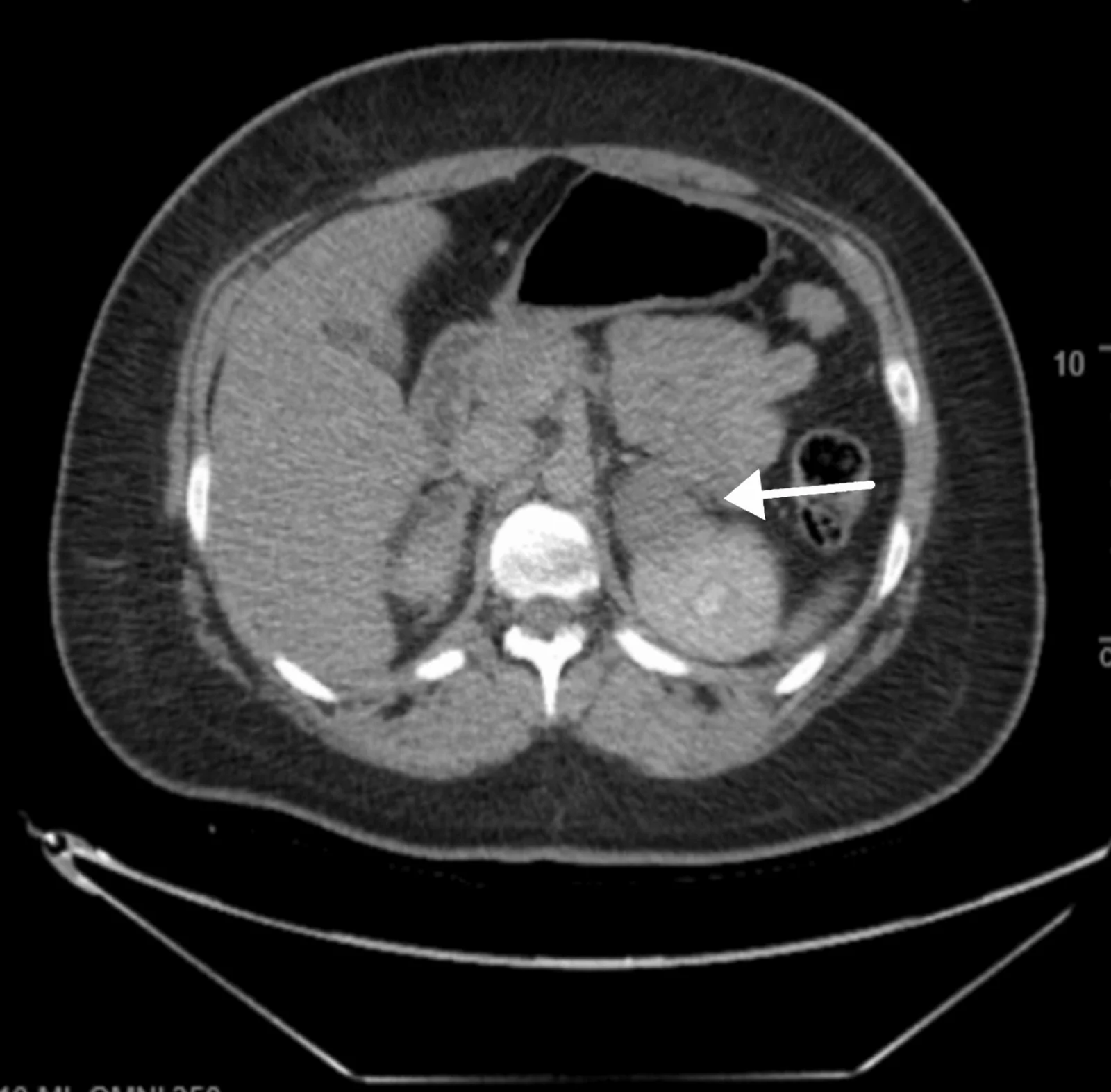
Axial CT view of the abdomen with IV contrast (the arrow points to the mass)

MIBG scintigraphy was performed, which showed bilateral functioning pheochromocytoma. Also, ultrasound of the thyroid was performed, which showed a right thyroid hypoechoic lesion measuring 1.3 x 1.6 x 2.2 cm, located at the lower pole, which could represent a thyroid versus parathyroid nodule (Figure 3).

**Figure 3:**
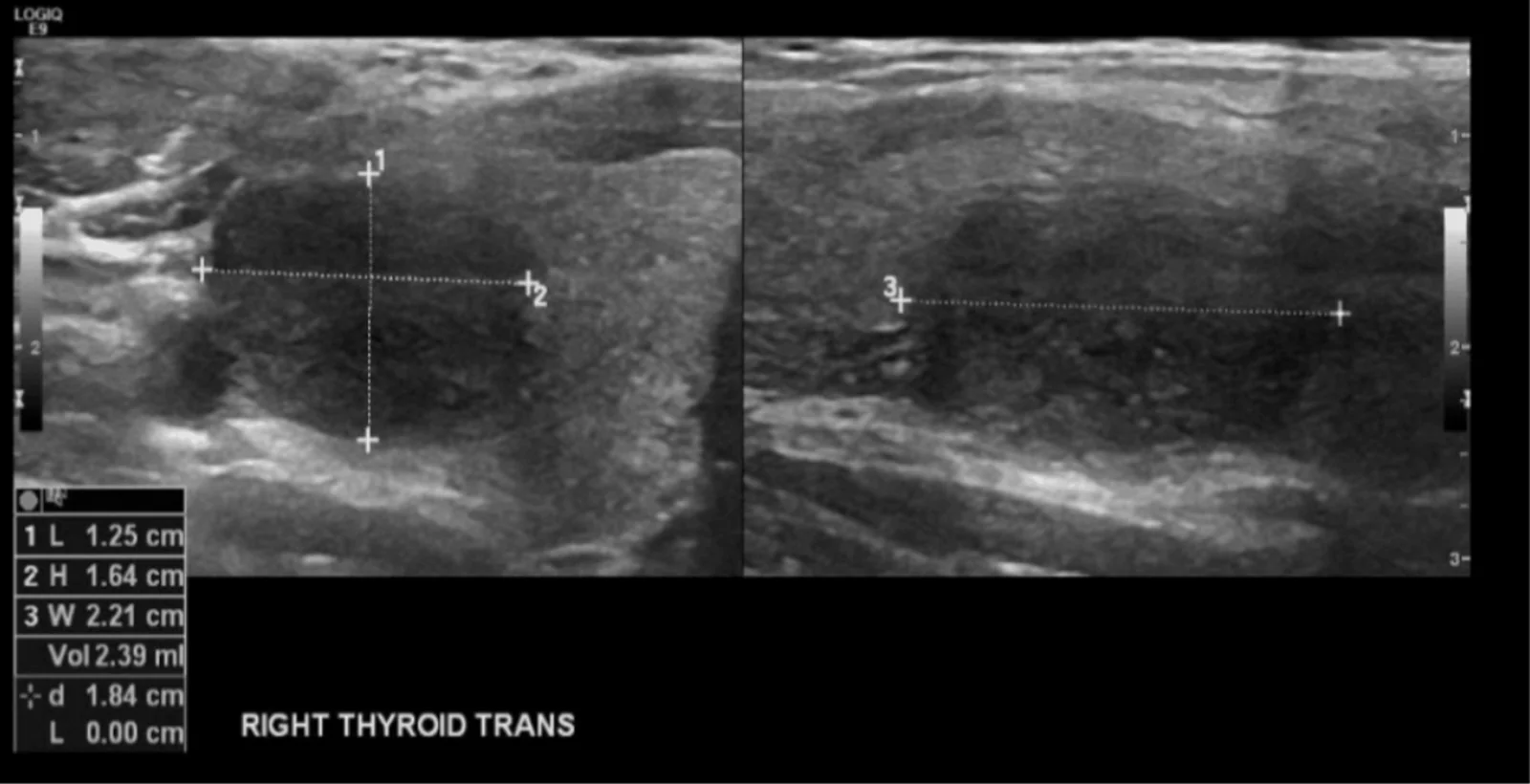
Ultrasound thyroid showing a right thyroid nodule

Parathyroid technetium-99m sestamibi (MIBI) scan was performed, which was negative for parathyroid hyperplasia or adenoma. TSH level and thyroid function tests were sent and resulted as follows: TSH 2.280 IU/L , T4 16.2 pmol/L, and T3 4.5 pmol/L. Parathyroid hormone (PTH) and calcium levels were measured, which were within normal limits. Fine-needle aspiration (FNA) biopsy was performed for the former nodule, which revealed medullary thyroid carcinoma. Calcitonin test was sent for follow-up purpose and resulted as 17 pg/mL. The patient was diagnosed with MEN 2 with medullary thyroid cancer (MTC) and bilateral pheochromocytoma. The endocrinology team was following the patient during admission and started preparing the patient for surgery with anti-hypertensive phenoxybenzamine 10 mg twice a day and then carvedilol 25 mg twice a day. After optimizing the patient's condition, she underwent bilateral laparoscopic retroperitoneal adrenalectomy. Her postoperative course was uneventful, and the patient was discharged home in stable condition. Histopathology showed a 6-cm left adrenal pheochromocytoma with involved margins and a 5-cm right adrenal pheochromocytoma with free margins.

Two months later, the patient was admitted under the care of the Breast and Endocrine Surgery Department, where she underwent total thyroidectomy with central and lateral neck dissection. Postoperatively, the patient had an uneventful course and was discharged on thyroxine with normal calcium and PTH levels. Histopathology showed bilateral multifocal MTC; the largest focus was on the right side, measuring 0.7 cm, with free margins and no lymphovascular invasion or extrathyroidal extension. The patient underwent genetic testing, which revealed a positive rearranged during transfection (RET) proto-oncogene mutation. Consequently, family members were advised to undergo genetic screening for the same mutation.

The patient was lost to follow-up during the COVID-19 pandemic. She returned to the endocrinology clinic in 2021 with complaints of headache and elevated blood pressure. Further investigations were arranged on an outpatient basis. An NM (nuclear medicine) MIBG scan was performed, which showed a slight interval progression in the activity and size of soft tissue lesion at the left adrenal bed, with the impression of recurrent/residual pheochromocytoma. Also, CT of the abdomen and pelvis and PET/CT scan were performed, which showed multiple nodules within the left adrenal bed with high metabolic activity on the PET scan, consistent with a recurrent pheochromocytoma lesion (Figures 4, 5).

**Figure 4:**
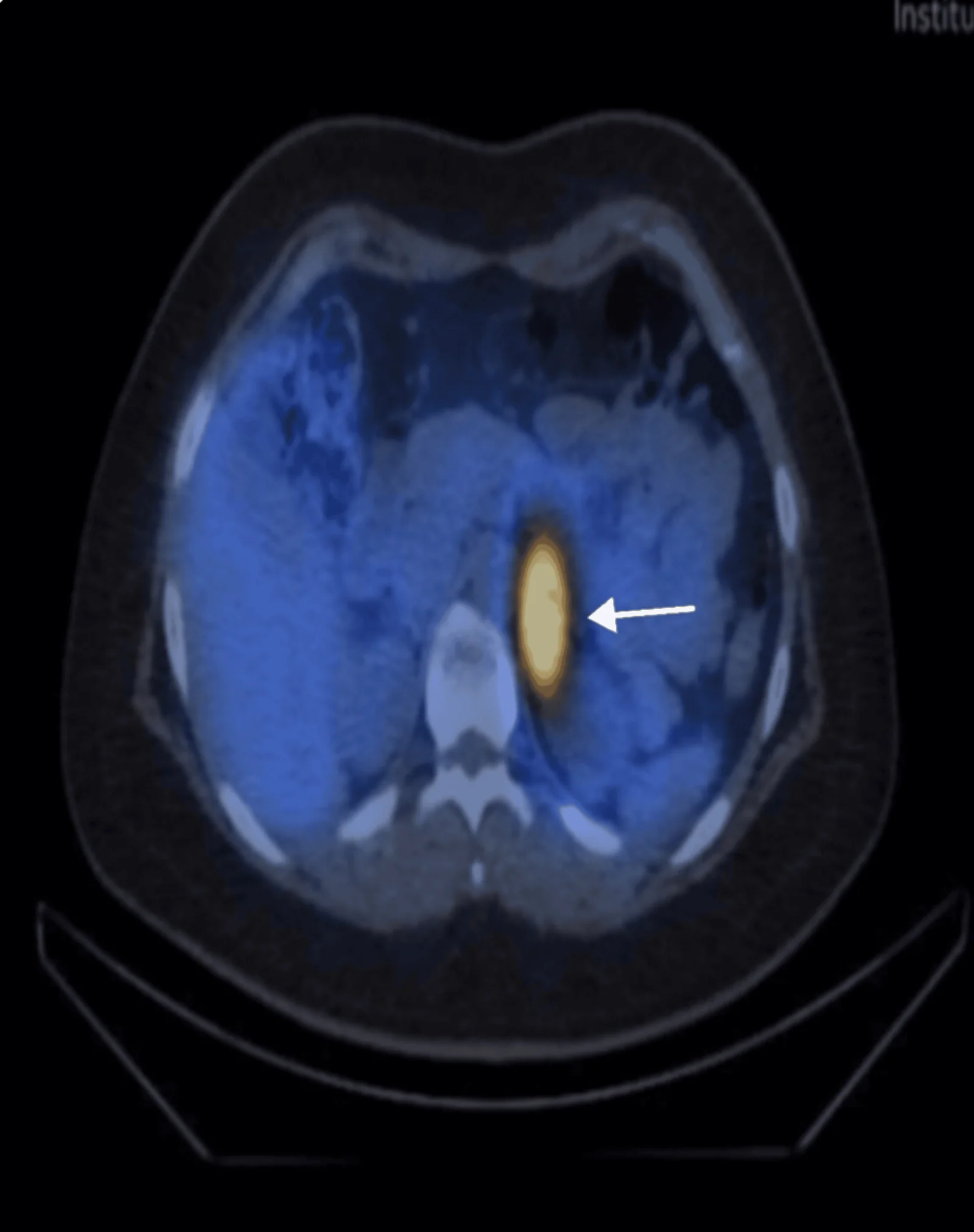
PET/CT showing an active left adrenal lesion with a high metabolic activity, which matched a previous MIBG scan, most likely representing residual or recurrent pheochromocytoma lesion (the arrow points to the mass).

**Figure 5:**
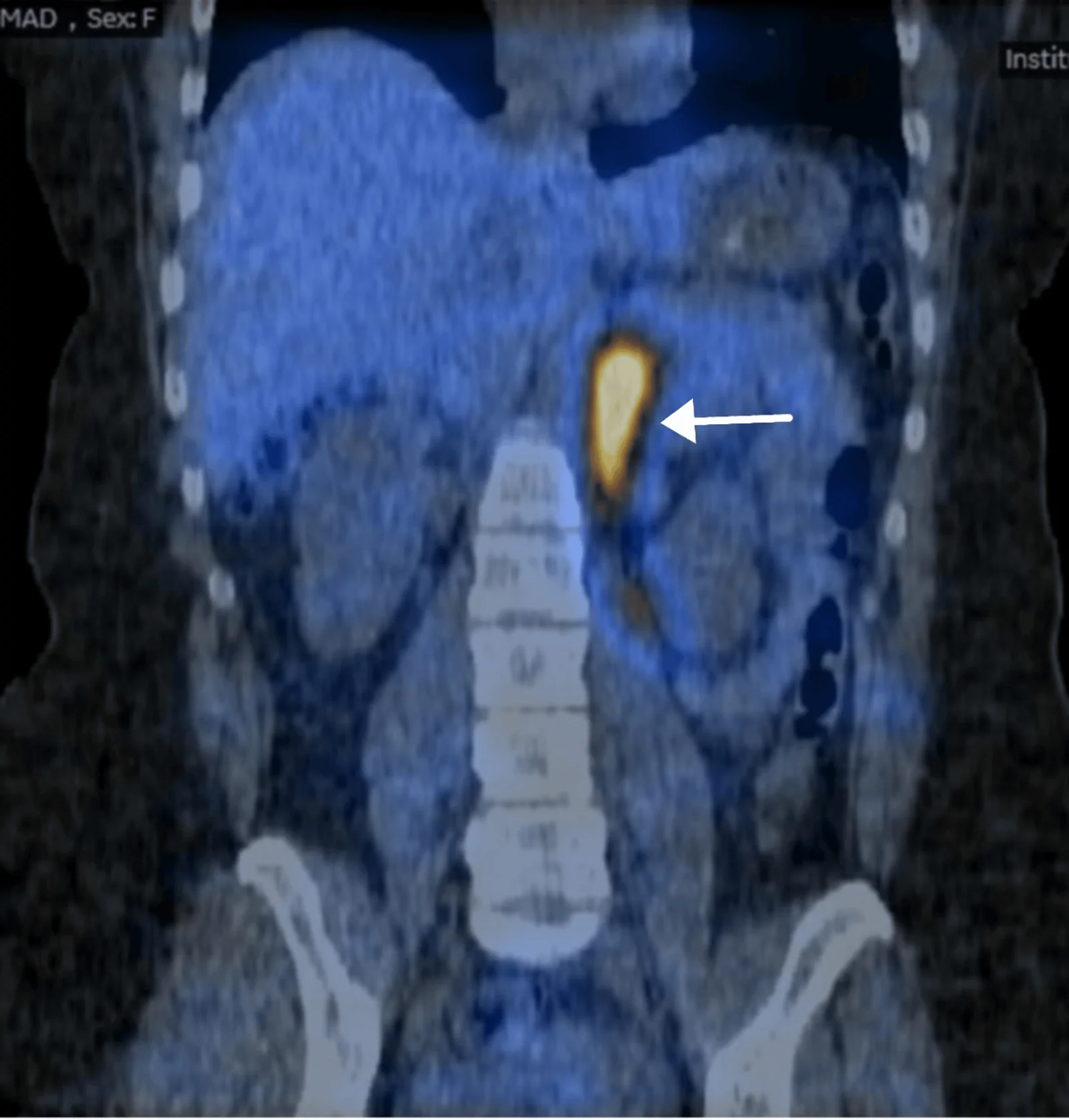
PET/CT showing an active left adrenal lesion with a high metabolic activity which matched a previous MIBG scan, most likely representing residual or recurrent pheochromocytoma lesion (the arrow points to the mass).

Laboratory investigation was repeated, which showed the following: adrenaline level of 0.977 nmol/L (reference range of less than or equal to 0.435 nmol/L), metanephrine level of 1,550 ng/L (reference range of less than 70 ng/L), noradrenaline level of 2,015 nmol/L (reference range of less than or equal to 2.89 nmol/L), normetanephrine level of 205 ng/L (reference range of less than 120 ng/L), and dopamine level of <0.650 nmol/L (reference range of less than or equal to 0.650 nmol/L). The case was discussed in the multidisciplinary team meeting, and it was planned to proceed with mass excision, cytoreductive surgery (CRS), and hyperthermic intraperitoneal chemotherapy (HIPEC).

After discussion with the patient and the family, they agreed on the surgical resection and refused the CRS and HIPEC. In 2023, the patient underwent laparoscopic exploration, which was converted to open exploration due to a finding of a retroperitoneal mass adherent to the renal veins, upper pole of the kidney, celiac axis, and diaphragm, without invading major vessels or the surrounding structures (Figure 6). A complete en bloc resection of the mass was performed with para-aortic node dissection. The histopathology results are shown in Table 2.

**Figure 6:**
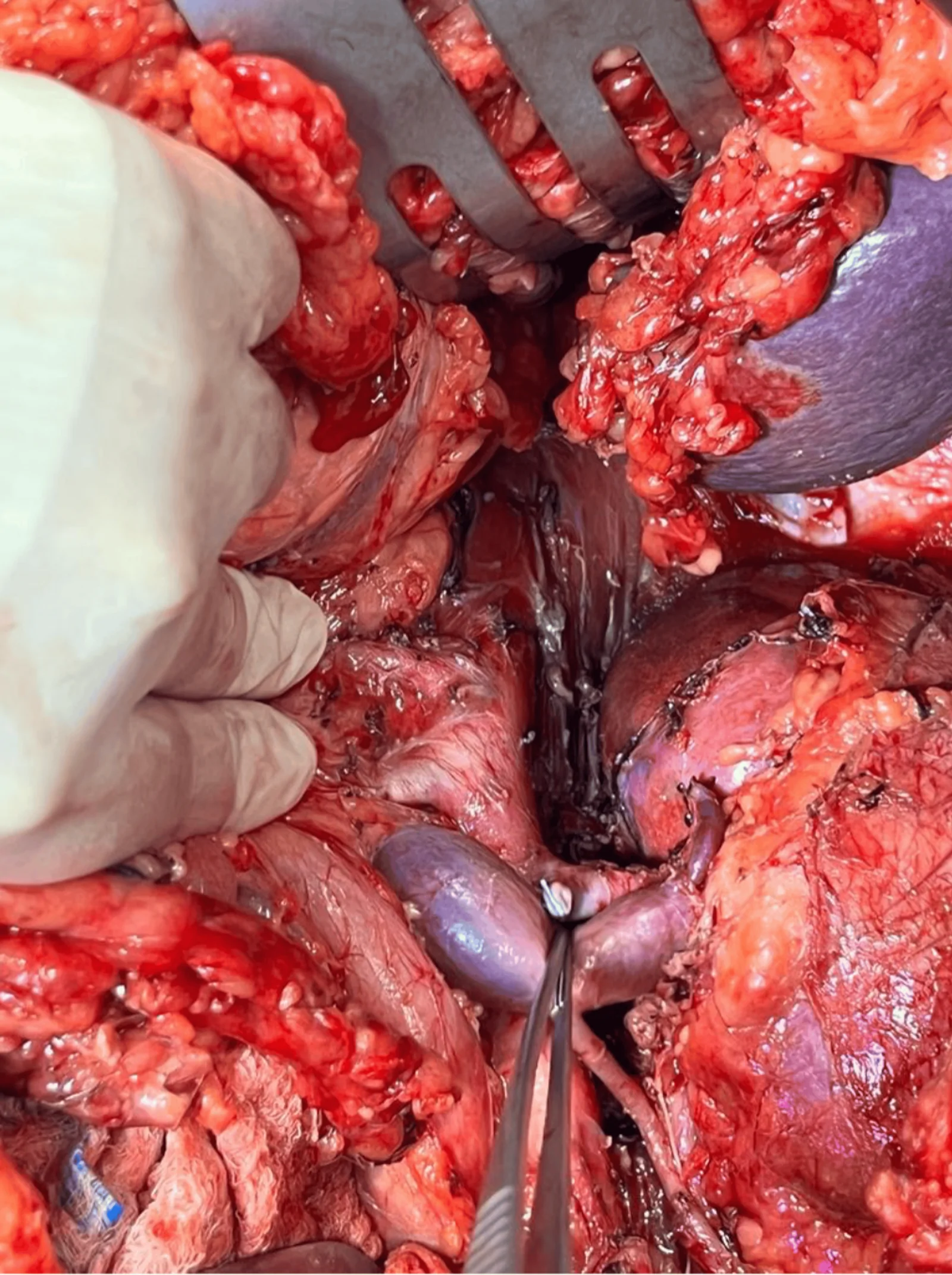
Intraoperative finding of the retroperitoneal mass adherent to the renal veins (picture taken after resection)

**Table 2:** Histopathology results

Histopathology result	Pheochromocytoma
Periadrenal adipose tissue invasion	Present
Skeletal muscle invasion	Present
Vascular invasion	Present in small lymphatic channels (multiple foci)
Capsular invasion	Present
Margins	Margin cannot be assessed (fragments)
Lymph nodes	19 reactive lymph nodes (0/19)

The patient was managed by multidisciplinary teams, and the histopathology and operative findings were discussed in the Tumor Board meeting. The patient was managed as a case of recurrent and malignant pheochromocytoma due to the Pheochromocytoma of the Adrenal Gland Scaled Score (PASS) of 4. The multidisciplinary team decided to treat the tumor bed with iodine-MIBG adjuvant radiotherapy. If the patient did not respond to radiotherapy, chemotherapy would then be considered. Therefore, she received adjuvant radiotherapy. The patient is on regular follow-up, whereas the latest CT scan performed in September 2024 was negative for recurrence.

## Discussion

MEN 2 comprises autosomal dominant neoplasm syndromes due to germline mutation in the RET proto-oncogene, which presents clinically as multiple neoplasms [6,7]. MEN 2 syndrome has clinical types 2A (MEN 2A) and 2B (MEN 2B). MEN 2 presents with four phenotypes, while classical MEN 2A presents with MTC in almost 100% of patients, pheochromocytoma in 50% of patients, and primary hyperparathyroidism in 10-20% [8,9]. MEN 2A may be associated with cutaneous lichen amyloidosis and Hirschsprung’s disease, while familial medullary thyroid carcinoma (MTC) represents another hereditary form of the disease [8-10]. MEN 2B is characterized by a 100% risk of developing MTC and a 30-50% incidence of pheochromocytoma. Primary hyperparathyroidism is rare, while benign ganglioneuromas may also occur [8,9,11,12].

MEN 2 syndrome is inherited in an autosomal dominant manner; a patient with a parent diagnosed of MEN 2 will have a risk of MEN 2A and MEN 2B of 95% and 50%, respectively. MEN syndrome is diagnosed when two or more of the characteristic associated neoplasms are present [4,8,9]. Genetic testing of the RET proto-oncogene gives a definitive diagnosis as it is positive in 98% of patients [11]. The American Thyroid Association recommends genetic testing for any patient with a first-degree relative with confirmed hereditary MTC, infants and children expressing the phenotype of MEN 2B or diagnosed with Hirschsprung disease, and patients diagnosed with cutaneous lichen amyloidosis. Total thyroidectomy is curative in MTC associated with MEN syndrome; therefore, annual screening and prophylactic thyroidectomy are advised to be performed in infants diagnosed with MEN 2B and in three-year-old children diagnosed with MEN 2A [8].

Pheochromocytoma is diagnosed using biochemical testing followed by radiological imaging. Biochemical evaluation involves measuring free plasma metanephrines and normetanephrines or 24-hour urinary metanephrines and normetanephrines. If the laboratory results are positive, then adrenal imaging is performed using CT, MRI, or MIBG scanning [3,4]. Assessing functional pheochromocytoma and controlling the blood pressure is a must prior to any surgical intervention. Therefore, adrenalectomy is a priority over thyroidectomy and parathyroidectomy to prevent hypertensive crisis intra-operatively [4]. In pheochromocytoma, surgical excision is the cornerstone, and in case of tumors less than 6 cm, laparoscopic excision is the preferable approach. Recurrence of pheochromocytoma post-excision is around 10%. Therefore, follow-up post-excision for a minimum of 10 years is advised [13-16].

Malignant pheochromocytoma is only 10% of all pheochromocytomas, although there are no histological criteria for the metastasis except the local or distant spread of the disease. Therefore, PASS was created to differentiate between benign and malignant pheochromocytoma; it is a multi-parameter score according to 12 histological features, where a cumulative score higher than 4 is considered malignant. In our case, the calculated PASS was 4, and therefore it was managed as malignant recurrent pheochromocytoma [14,15]. In case of malignant pheochromocytoma, ^131^I MIBG radiation therapy is recommended in tumors with positive 123 MIBG uptake. CRS with HIPEC is another option. We found a case report published in 2024 where a 25-year-old female patient with recurrent pheochromocytoma was managed with CRS and HIPEC; she was followed for six years with no evidence for recurrence [16]. However, the use of CRS and HIPEC in recurrent pheochromocytoma is still controversial due to a lack of studies and clinical trials regarding the efficacy of CRS with HIPEC in recurrent disease [15].

In MTC, the American Thyroid Association recommends total thyroidectomy with central neck dissection, while lateral neck dissection is recommended in patients with clinically palpable lymphadenopathy or positive lymphadenopathy on ultrasonography. If the patient is at a high risk for loss to follow-up, then modified radical neck dissection is advised [4]. In cases diagnosed with metastatic MTC, kinase inhibitors can be considered [4-13]. If the patient is diagnosed with primary hyperparathyroidism, surgical resection of one or more of the parathyroid glands is indicated [4].

## Conclusions

MEN 2 syndrome is inherited in an autosomal dominant manner, which is diagnosed by the presence of two or more of the associated neoplasms. Pheochromocytoma is one of the neoplasms associated with MEN syndrome. Pheochromocytoma is diagnosed using biochemical and radiological investigations by measuring metanephrines and normetanephrines in the plasma and urine, as well as by adrenal imaging such as CT scan. In pheochromocytoma, the surgical excision is the cornerstone, with a recurrence rate of 10%; therefore, follow-up post-excision is recommended.
